# Poly(ADP-Ribose) Polymerases-Inhibitor Talazoparib Inhibits Muscle Atrophy and Fatty Infiltration in a Tendon Release Infraspinatus Sheep Model: A Pilot Study

**DOI:** 10.3390/metabo14040187

**Published:** 2024-03-26

**Authors:** Maurits G. L. Olthof, Anita Hasler, Paola Valdivieso, Martin Flück, Christian Gerber, Rieke Gehrke, Karina Klein, Brigitte von Rechenberg, Jess G. Snedeker, Karl Wieser

**Affiliations:** 1 Department of Orthopaedics, Balgrist, University of Zurich, Forchstrasse 340, 8008 Zurich, Switzerland; anita.hasler@balgrist.ch (A.H.); christian.gerber@balgrist.ch (C.G.); jess.snedeker@balgrist.ch (J.G.S.); karl.wieser@balgrist.ch (K.W.); 2 Laboratory for Muscle Plasticity, Department of Orthopedics, Balgrist Campus, University of Zurich, Forchstrasse 340, 8008 Zurich, Switzerland; paola.valdivieso@yahoo.com (P.V.); martin.flueck@tutanota.com (M.F.); 3 Musculoskeletal Research Unit, Center for Applied Biotechnology and Molecular Medicine, Equine Department, Vetsuisse Faculty, Winterthurerstrasse 190, 8057 Zurich, Switzerland; r.gehrke@fu-berlin.de (R.G.); kklein@vetclinics.uzh.ch (K.K.); brigitte.vonrechenberg@uzh.ch (B.v.R.); 4 Institute for Biomechanics, ETH Zurich, Gloriastrasse 37/39, 8092 Zürich, Switzerland

**Keywords:** rotator cuff rupture, experimental sheep model, muscle atrophy, fatty infiltration, PARP inhibition

## Abstract

Structural muscle changes, including muscle atrophy and fatty infiltration, follow rotator cuff tendon tear and are associated with a high repair failure rate. Despite extensive research efforts, no pharmacological therapy is available to successfully prevent both muscle atrophy and fatty infiltration after tenotomy of tendomuscular unit without surgical repair. Poly(ADP-ribose) polymerases (PARPs) are identified as a key transcription factors involved in the maintenance of cellular homeostasis. PARP inhibitors have been shown to influence muscle degeneration, including mitochondrial hemostasis, oxidative stress, inflammation and metabolic activity, and reduced degenerative changes in a knockout mouse model. Tenotomized infraspinatus were assessed for muscle degeneration for 16 weeks using a Swiss Alpine sheep model (n = 6). All sheep received daily oral administration of 0.5 mg Talazoparib. Due to animal ethics, the treatment group was compared with three different controls from prior studies of our institution. To mitigate potential batch heterogeneity, PARP-I was evaluated in comparison with three distinct control groups (n = 6 per control group) using the same protocol without treatment. The control sheep were treated with an identical study protocol without Talazoparib treatment. Muscle atrophy and fatty infiltration were evaluated at 0, 6 and 16 weeks post-tenotomy using DIXON-MRI. The controls and PARP-I showed a significant (control *p* < 0.001, PARP-I *p* = 0.01) decrease in muscle volume after 6 weeks. However, significantly less (*p* = 0.01) atrophy was observed in PARP-I after 6 weeks (control 1: 76.6 ± 8.7%; control 2: 80.3 ± 9.3%, control 3: 73.8 ± 6.7% vs. PARP-I: 90.8 ± 5.1% of the original volume) and 16 weeks (control 1: 75.7 ± 9.9; control 2: 74.2 ± 5.6%; control 3: 75.3 ± 7.4% vs. PARP-I 93.3 ± 10.6% of the original volume). All experimental groups exhibited a statistically significant (*p* < 0.001) augmentation in fatty infiltration following a 16-week period when compared to the initial timepoint. However, the PARP-I showed significantly less fatty infiltration (*p* < 0.003) compared to all controls (control 1: 55.6 ± 6.7%, control 2: 53.4 ± 9.4%, control 3: 52.0 ± 12.8% vs. PARP-I: 33.5 ± 8.4%). Finally, a significantly (*p* < 0.04) higher proportion and size of fast myosin heavy chain-II fiber type was observed in the treatment group. This study shows that PARP-inhibition with Talazoparib inhibits the progression of both muscle atrophy and fatty infiltration over 16 weeks in retracted sheep musculotendinous units.

## 1. Introduction

Rotator cuff tears are highly prevalent and represent the leading cause of shoulder dysfunction and pain [1]. They often require surgical repair to improve clinical outcomes; however, failure rates in achieving structural repair range from 13% to 94% [2]. Chronic rotator cuff tears are characterized by degenerative changes in the musculotendinous unit, encompassing structural, architectural and functional alterations. While architectural and functional changes can be partially reversed following successful repair, structural changes remain irreversible [3]. These structural changes primarily involve muscle atrophy, fibrosis and fatty infiltration, all of which exert a direct impact on the success and clinical outcome of the repair procedure [4]. Notably, muscle atrophy and fatty infiltration have been identified as independent predictors of unsuccessful surgical repair.

Hence, various pharmacological interventions have been explored to impede muscle degeneration and promote muscle regeneration in the context of rotator cuff tears. Nevertheless, there are currently no clinical therapies available specifically targeting the inhibition of fatty infiltration and muscle atrophy. Through investigations using preclinical models, a few potential candidates have been identified that can impact muscle degeneration. For instance, Tamoxifen, a competitive inhibitor of estrogen receptors, demonstrated the ability to inhibit muscular atrophy and inflammation following 16 weeks of musculotendinous retraction in mice; however, it was ineffective in inhibiting fatty infiltration [5]. Conversely, the PDGFR inhibitor Imatinib exhibited suppression of fatty infiltration only in a mouse rotator cuff tear model following 4 weeks of musculotendinous unit retraction [6]. Furthermore, Nandrolone exhibited potent long-term inhibition of fatty infiltration in a large sheep musculotendinous retraction model after 16 weeks; however, it did not effectively reduce muscular atrophy [7].

Poly(ADP-ribose) polymerases (PARPs) play a crucial role as transcription factors in maintaining cellular homeostasis. The primary contributors to PARP activity are PARP-1 (85–90%) and PARP-2 (10–15%) [8]. These factors are involved in various intracellular pathways affecting muscle degeneration, including mitochondrial function, oxidative stress, inflammation and metabolic activity. PARPs regulate processes like mitochondrial dysfunction-induced cell death [9], the production of inflammatory cytokines [10,11,12,13,14], Peroxisome proliferator-activated receptor gamma (PPAR-γ)-mediated adipogenesis [15,16] and the control of glucose homeostasis [16]. Consequently, these transcription factors are implicated in pathways associated with muscle atrophy, muscle fatty infiltration and the inhibition of muscle regeneration. Deletion of these transcription factors is therefore linked to a reduction in the proinflammatory response of innate immune system cells, endothelial cells and fibroblasts [8,10]. Moreover, their absence inhibits adipogenesis [15,16] and enhances insulin sensitivity by promoting mitochondrial biogenesis [16].

A preclinical study utilizing a PARP-1 knockout mouse model demonstrated the potential of PARP inhibition in preventing muscle degeneration. The PARP-1 knockout mice showed reduced early inflammation, muscle atrophy and fatty infiltration in the rotator cuff muscle after tenotomy and denervation [17]. Furthermore, pharmacological PARP inhibition using MRL-45696, a dual PARP-1/PARP-2 inhibitor derived from Niraparib, exhibited promising outcomes for muscle regeneration in mice. MRL-45696 treatment led to enhanced skeletal muscle mitochondrial function, as evidenced by improved oxidative mitochondrial respiration, increased running capacity and better insulin sensitivity compared to the control group [18]. Various PARP inhibitors, such as Oleparib, Rucaparib, Niraparib and Talazoparib, have received FDA approval for safe and effective treatment of ovarian and breast cancer [19]. Among these, Talazoparib demonstrated the highest potency as a dual PARP-1/PARP-2 inhibitor, with a PARP-trapping potential approximately 100-times greater than that of other PARP inhibitors [20]. Due to this high efficacy, Talazoparib was chosen for investigating the effects of PARP inhibition on muscle degeneration.

In our previous research, we successfully demonstrated the reproducibility of clinical musculotendinous changes resulting from rotator cuff tendon failure [3,7,21,22,23]. This validation was achieved using a preclinical sheep rotator cuff model [3,7,21,22,23]. Building upon this foundation, we now intend to conduct a pilot study to explore the impact of PARP inhibition on preventing muscle degeneration in the sheep rotator cuff muscle. Our hypothesis is that PARP inhibition will be associated with a reduction in both muscle atrophy and fatty infiltration of the rotator cuff. This study aims to provide valuable insights into the potential therapeutic benefits of PARP inhibition for mitigating muscle degeneration in this specific context.

## 2. Materials and Methods

### 2.1. Experimental Design

A chronic rotator cuff tear model, characterized by musculotendinous retraction, fatty infiltration, muscle atrophy and impaired muscle function, was established using a validated sheep infraspinatus (ISP) tenotomy model. The protocol for this study was approved by the investigational review board and local Swiss federal authorities (Approval No. 130/19) and was based on previous research conducted by our group [3,7,21,22,23]. In this pilot study, the therapeutic effect of pharmacological PARP-inhibitory therapy using Talazoparib was investigated in tenotomized infraspinatus muscles over a 16-week period without tendon reconstruction. To ensure sufficient statistical power, a power analysis was conducted based on our previous sheep studies, suggesting that a sample size of n = 6 per group would be required to detect a relevant difference of at least 20% at an alpha of 0.05 and a standard deviation of 6, with a power of 80%. In compliance with the ethical principles of animal experimental research—Replace, Reduce and Refine—and considering the nascent understanding of the pharmacological effects of PARP inhibition on muscle degeneration at the time our study commenced, we opted for a pilot study design. This design involved a comparison between the treatment group receiving oral Talazoparib immediately post-tenotomy (PARP inhibition group, PARP-I, n = 6) and three distinct negative control groups (n = 6 each), each derived from previously conducted infraspinatus tenotomy studies in sheep. To mitigate potential batch heterogeneity, PARP-I was evaluated in comparison with three distinct control groups using the same protocol without treatment. The entire study (PARP-I and control groups) was conducted at the same research facility, using female Swiss Alpine Sheep (PARP-I: age 26.0 ± 0 months, weight 61.8 ± 3.2 kg, Control 1: age 23.2 ± 1.0 months, weight 45.3 ± 4.8 kg, Control 2: age 24.0 ± 1.9 months, weight 48.4 ± 3.8 kg, Control 3: 29.4 ± 4.8 months, weight 55.3 ± 7.7 kg), with identical nutrition and mobility protocols. The surgical procedures were performed by the same surgeon under uniform conditions. One negative control group (n = 6, control group 1) was utilized for muscle architecture and histological analysis. Muscle degeneration was assessed using Magnetic Resonance Imaging-Dixon (MRI-DIX) at 0, 6 and 16 weeks, and histological analysis was performed at 16 weeks, following the exact same standardized protocols used in our previous studies [7].

### 2.2. Surgical Technique

All surgical procedures were carried out on the right shoulder of each sheep, while the left shoulder was used as a control. A 15 cm curved incision, positioned 2 cm caudal to the scapular spine, was made to release the infraspinatus (ISP) tendon. An osteotomy of the greater tuberosity measuring 20 × 10 × 10 mm was performed using an oscillating saw. Prior to the tendon release, a biopsy of the ISP muscle was taken using a 5 mm diameter Bergstrom needle from Dixons Surgical Instruments LTD., Wickford, UK. The tendon stump, along with the bone chip, was secured using Fiberwire No. 5 sutures, employing two figure-of-8 stitches (Arthrex Inc., Naples, FL, USA), passing through a 1.8 mm drill hole at the center of the bone chip. Subsequently, the tendon and bone chip, with the sutures intact, were enclosed in a silicone tube (Silicone Penrose drain tube, 12 mm diameter; Fortune Medical Instruments, Taipei, Taiwan) to prevent spontaneous reattachment due to scar tissue. The wound was then closed.

Immediately after surgery, MRI-DIX scans were conducted on both shoulders while the animal remained under general anesthesia. At 6 weeks and before sacrifice at 16 weeks, additional MRI-DIX scans were repeated with the animal under general anesthesia. Subsequently, both the tenotomized and contralateral ISP musculotendinous units were biopsied, and after euthanasia, they were meticulously dissected for further analysis.

### 2.3. Anesthesia and Euthanasia

For all surgical interventions and radiographic assessments, the sheep were anesthetized following a uniform protocol. Sedation was initiated from 30 to 60 min prior to the induction of general anesthesia by intramuscular administration of 5 μg/kg medetomidine (Domitor; Orion Pharma, Turku, Finland), and analgesia was provided through an intravenous dose of 4 mg/kg carprofen (Rimadyl; Pfizer, New York, NY, USA). Anesthesia induction was achieved with an intravenous injection of 1% propofol (Diprivan; AstraZeneca, London, UK) at a dosage from 2 to 4 mg/kg. Maintenance of general anesthesia was carried out using 1% isoflurane (Forane; Abbott AG, Baar, Switzerland) in oxygen. Prophylactic tetanus antiserum (Tetanus Serum; Veterinaria AG, Zurich, Switzerland) was administered immediately prior to surgery. For analgesic purposes, carprofen was given subcutaneously on the day of surgery and during the first three days postoperatively, along with 7 mg/kg gentamicin (Streuli, Uznach, Switzerland) and 30,000 IU/kg penicillin G (Hoechst, Frankfurt, Germany) administered intravenously twice daily. The sheep were required to fast for 24 h before the procedure but were otherwise allowed food and water ad libitum. After 16 weeks, the animals were euthanized in deep anesthesia using pentobarbital (150 mg/kg body weight).

### 2.4. Pharmacological Treatment

The treatment group (PARP-I) comprised six sheep, each of which received a daily oral dose of 0.5 mg Talazoparib (equating to 0.008 ± 0.0004 mg/kg/day) (Medkoo Biosciences, Durham, NC, USA). This administration was facilitated using a syringe by an animal care specialist to ensure complete consumption. The Talazoparib was dissolved in a solution consisting of 10% Dimethyl sulfoxide (DMSO), 40% polyethylene glycol (PEG) 400, 5% Tween 80 and 45% phosphate-buffered saline (PBS) and was administered immediately following tendon release. The selection of the 0.5 mg dose was based on a trial approach since dose-response curves for Talazoparib in sheep were not available at the time of the study. As for the three control groups, the sheep did not receive any pharmacological treatment during the course of the study.

### 2.5. Radiological Assessment of Structural Muscular Changes

The MRI-DIX scans were conducted following a well-established protocol [7]. The scans were performed with the sheep in a supine position using a 3-Tesla MRI system equipped with a dedicated receive-only extremity coil (Philips Ingenia 3T with dStream body coil Solution, Philips AG, Amsterdam, The Netherlands). To ensure accurate imaging, the scapular spines of both shoulders were properly positioned in the imaging plane, allowing for transverse sections perpendicular to the glenoid cavity. The pulse sequence employed included T1-weighted turbo spin echo (TSE) transverse, proton density-weighted TSE transverse, T1-weighted TSE coronal, T2-weighted spectral presaturation with inversion recovery (SPIR) coronal and the Dixon method transverse in-phase, transverse out-phase, transverse water-only and transverse fat-only images.

For the evaluation of muscle volume, T1-weighted transverse images were utilized. The cross-sectional area along the entire length of both infraspinatus (ISP) muscles, including the central tendon (excluding the bone chip), was delineated, and the corresponding muscle volume was computed. To assess the fat fraction (FF), multiple cross-sectional areas of the muscle at the level of the central tendon were analyzed. Subsequently, the FF was calculated based on the signal intensities obtained from fat-only images (Dixon transverse fat-only (DIXON-FAT)) and water-only images (Dixon transverse water only (DIXON-WATER)) using the following algorithm: FF = DIXON-FAT/(DIXON-FAT + DIXON-WATER). For the evaluation of musculotendinous retraction, the distance between the bone chip and its original insertion site was measured in one complete transverse cross-sectional area at the level of the central tendon of the infraspinatus muscle. Additionally, the pennation angle and fascicle length were assessed on T1-weighted transverse images using a method previously described [24].

The MRI-DIX scans of both the control and treatment groups were reviewed by a single observer, employing a well-established method with excellent intraobserver reliability, utilizing the DICOM viewer OsiriX v 5.6 32-bit [7]. The precise validated methodology developed by our research group was transferred from the previous observer to the new observer, and an interobserver analysis was conducted by comparing the reevaluated data from the control group with measurements previously performed.

### 2.6. Histological Assessment

After harvesting, the biopsies were promptly embedded in Cryomolds (Sakura Finetek, Sysmex Suisse AG, Horgen, Switzerland) using Tissue-Tek O.C.T compound (Sakura Finetek, Sysmex Suisse AG, Horgen, Switzerland) and rapidly frozen in −80 °C-cold 2-methylbutane (MERCK, Darmstadt, Germany) using a Snapfrost device (Excilone, Elancourt, France). The frozen biopsies were stored at −80 °C until further analysis.

To investigate muscle fiber composition and lipid content, 12 μm cryosections were cut perpendicular to the major fiber axes of most fiber profiles using a cryostat (CM3050S; Leica Biosystems, Wetzlar, Germany). These sections were then mounted on cryo-slides and stored at −80 °C until further use.

The area percentage of slow-, fast- and hybrid-type muscle fibers was determined using double immunofluorescent staining for the two main myosin heavy-chain types, following a previously described methodology [25]. In brief, the fixed sections were initially incubated with goat serum and then exposed to both primary antibodies (fast-type myosin antibody, No. ab91506; Abcam, Cambridge, UK, and slow-type myosin antibody, No. MAB1628; Millipore Corp., Temecula, CA, USA) in a bovine serum albumin-phosphate-buffered saline (BSA-PBS) buffer. After thorough washing, the samples were incubated with the secondary antibodies Alexa Fluor 555-coupled anti-mouse and Alexa Fluor 488-coupled goat anti-rabbit antibody (No. A21425 and No. A11017, Thermo Fisher Life Technologies, Waltham, MA, USA). Subsequently, the nuclei were counterstained with Hoechst 33342 (No. 62249, Thermo Fisher Life Technologies, Waltham, MA, USA), and the sections were embedded in fluorescence mounting medium (Dako, Agilent Technologies, Santa Clara, CA, USA). The number and mean cross-sectional areas (MCSA) of slow (Myosin Heavy Chain (MHC)-I), fast (MHC-IIa, MHC IIx/d, MHC IIb), and slow/fast hybrid fibers were assessed using a previously described semi-quantitative method [25].

Muscle lipid percentage was determined using oil red O staining, as previously described [25]. Briefly, cryosections were thawed and fixed in 4% paraformaldehyde, stained with a working solution of oil red O (0.3 g, VWR international, Radnor, PA, USA) per 100 mL isopropanol (MERCK, Darmstadt, Germany) and counterstained with hematoxylin (Artechemis, Zofingen, Switzerland). Similar to the fiber-type analysis, the entire stained section was recorded for analysis, and the percentage of cross-sectional area representing the red signal was assessed using a macro with ImageJ software, version 2.1.0/1.53t/ Java 1.8.0_202. 

### 2.7. Statistics

Interobserver reliability was assessed using intraclass correlation coefficients (ICC). Data were subjected to statistical analysis using repeated measurements ANOVA with a Benjamini Hochberg post-hoc test to evaluate time and treatment effects. Parametric histological data were analyzed using an independent *t*-test. The statistical analyses were performed using SPSS for Windows version 25 (SPSS Inc., Chicago, IL, USA). All tests were two-tailed, and a significance level of *p* < 0.05 was considered statistically significant.

## 3. Results

During the follow-up period, no complications were observed in any of the sheep, and all collected data were deemed suitable for further analysis. The animals were under meticulous daily clinical observation and exhibited overall good health throughout the study with an increased weight of 65.9 ± 2.4 kg after 16 weeks.

Interobserver analysis of muscle volume demonstrated a strong correlation, with an interclass correlation coefficient (ICC) of 0.83, indicating reliable and consistent measurements. Similarly, measurements of fatty infiltration using MRI-DIX exhibited an excellent interobserver correlation, with an ICC of 0.99, further validating the accuracy and consistency of the data obtained from the imaging analysis.

### 3.1. Radiological Measurements

#### 3.1.1. Muscle Volume

**Figure 1:**
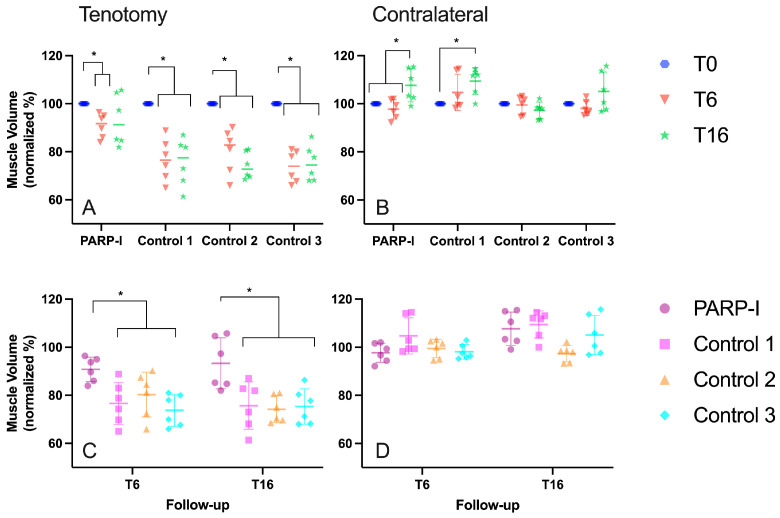
Muscle volume, normalized to the initial volume (T0) of the right (tenotomy **A**,**C**) and left (contralateral **B**,**D**) infraspinatus muscles in sheep, illustrating the difference between groups (**C**,**D**) and the effect of time (**A**,**B**). The treatment group, denoted as PARP-I, received the PARP inhibitor Talazoparib. An asterisk (*) indicates a statistically significant difference with a *p*-value of <0.05. The timepoint 6 weeks post-tenotomy is represented by T6, while T16 denotes the timepoint 16 weeks post-tenotomy.

The controls and PARP-I showed a significant (control *p* < 0.001, PARP-I *p* = 0.01) decrease in muscle volume after 6 weeks (Figure 1A). However, significantly less (*p* = 0.01) atrophy was observed in PARP-I after 6 weeks (control 1: 76.6 ± 8.7%; control 2: 80.3 ± 9.3%, control 3: 73.8 ± 6.7% vs. PARP-I: 90.8 ± 5.1% of the original volume) and 16 weeks (control 1: 75.7 ± 9.9; control 2: 74.2 ± 5.6%; control 3: 75.3 ± 7.4% vs. PARP-I 93.3 ± 10.6% of the original volume) (Figure 1C).

Concerning the intact left infraspinatus muscle, both the PARP-I and control group 1 demonstrated a significant (*p* < 0.03) augmentation in muscle volume following a 16-week period, as compared to the initial timepoint (Figure 1B). No significant differences were observed between all groups for all timepoints (Figure 1D).

#### 3.1.2. Fatty Infiltration

**Figure 2:**
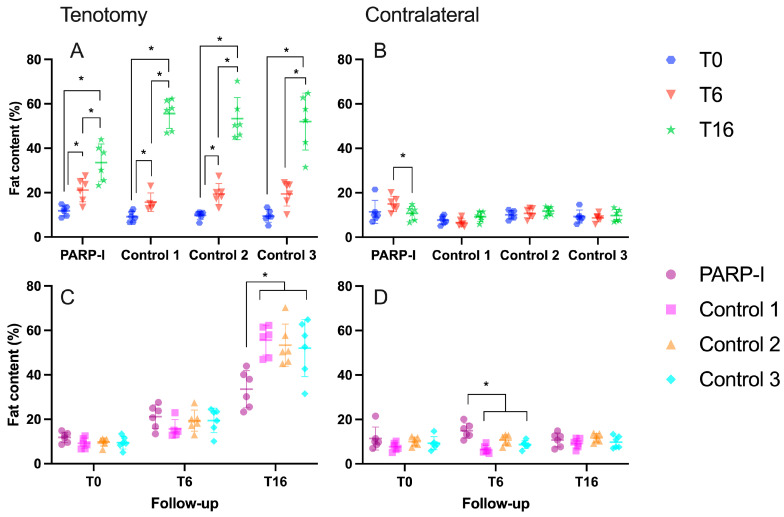
MRI-Dixon fat content (%) of the right (tenotomy **A**,**C**) and left (contralateral **B**,**D**) infraspinatus muscles in sheep, illustrating the difference between groups (**C**,**D**) and the effect of time (**A**,**B**). The treatment group, denoted as PARP-I, received the PARP inhibitor Talazoparib. An asterisk (*) indicates a statistically significant difference with a *p*-value of <0.05. The timepoint 6 weeks post-tenotomy is represented by T6, while T16 denotes the timepoint 16 weeks post-tenotomy.

All experimental groups exhibited a statistically significant (*p* < 0.001) augmentation in fatty infiltration following a 16-week period when compared to the initial timepoint, as illustrated in Figure 2A. However, the PARP-I showed significantly less fatty infiltration (*p* < 0.003) compared to all controls (control 1: 55.6 ± 6.7%, control 2: 53.4 ± 9.4%, control 3: 52.0 ± 12.8% vs. PARP-I: 33.5 ± 8.4%) (Figure 2C).

In relation to the contralateral intact infraspinatus muscle, a statistically significant (*p* = 0.04) reduction in fatty infiltration was observed for the PARP-I group between the 6-week and 16-week timepoints. No significant time-related effects were detected in the control groups, as depicted in Figure 2B. Although a significantly higher level of fatty infiltration was observed in the PARP-I group compared to the control groups after 6 weeks (control 1: 6.5 ± 1.8%, control 2: 10.7 ± 2.3%, control 3: 8.7 ± 1.8% vs. PARP-I: 15.0 ± 3.4%) with a *p*-value of less than 0.03, no significant differences were found among all groups after 16 weeks (control 1: 9.3 ± 2.2%, control 2: 11.8 ± 1.9%, control 3: 9.8 ± 2.7% vs. PARP-I: 10.8 ± 3.1%), as depicted in Figure 2D.

#### 3.1.3. Muscle Architecture

**Figure 3:**
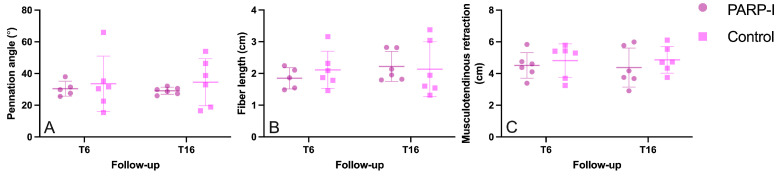
Musculotendinous retraction (**A**), muscle fiber pennation angle (**B**) and muscle fiber length (**C**) of tenotomized sheep infraspinatus muscles over time. The treatment group, denoted as PARP-I, received the PARP inhibitor Talazoparib. The timepoint 6 weeks post-tenotomy is represented by T6, while T16 denotes the timepoint 16 weeks post-tenotomy.

There were no statistically significant differences observed between the PARP-I group and the control group in terms of muscle retraction, pennation angle and fiber length following both 6 and 16 weeks of infraspinatus muscle retraction, as depicted in Figure 3A–C.

### 3.2. Histology

**Figure 4:**
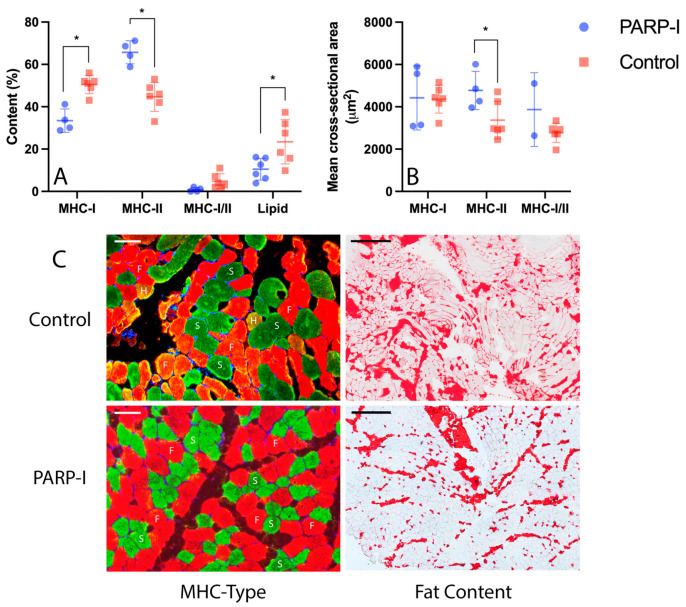
Histological analysis of supraspinatus muscle biopsies 16 weeks post-tenotomy: distribution of myosin heavy-chain (MHC) muscle fiber types and histological fat content in percentages (**A**), with the mean cross-sectional area of fibers presented in µm^2^ (**B**). Panel (**C**) showcases representative sections stained with double immunofluorescence for MHC typing (MHC-type) alongside Oil Red O for lipid content (fat content). S denotes slow muscle fibers, F indicates fast muscle fibers and H marks hybrid fast/slow fibers. Fat is visualized in red by Oil Red O staining. Scale bars are set at 100 µm for MHC immunofluorescence and 500 µm for Oil Red O lipid staining. The treatment group, denoted as PARP-I, received the PARP inhibitor Talazoparib. The control group displays the histological characteristics of the infraspinatus muscle 16 weeks after tenotomy without any treatment. An asterisk (*) indicates a statistically significant difference with a *p*-value of <0.05.

Two muscle biopsies were excluded from the analysis of MHC muscle fiber types due to suboptimal tissue quality resulting from a technical malfunction during tissue processing. Figure 4C presents an illustrative example of the MHC immunofluorescent staining and oil red O staining for both the PARP-I and control group.

In terms of histology from the 16-week tenotomized supraspinatus muscles, significant differences were observed between the PARP-I and control groups. The PARP-I group exhibited a significantly (*p* < 0.001) lower percentage of slow MHC-I fibers compared to the control group (PARP-I: 33.5% ± 5.5, control: 50.5% ± 4.3) (Figure 4A). Conversely, a significantly (*p* < 0.001) higher percentage of fast MHC-II fibers was observed in the PARP-I group (PARP-I: 65.7% ± 5.4, control: 44.7% ± 6.8) (Figure 4A). Hybrid fibers were present in both groups, with a non-significantly (*p* = 0.06) higher percentage of hybrid fibers in the control group compared to PARP-I (PARP-I: 4.8 ± 3.5, control: 0.8 ± 1) (Figure 4A). Regarding fatty infiltration, the PARP-I group demonstrated significantly (*p* = 0.03) lower fat content compared to the control group at the 16-week timepoint (PARP-I: 10.5% ± 5.1, control: 23.5% ± 10.5) (Figure 4A). Additionally, a significantly (*p* = 0.04) higher mean cross-sectional area of fast MHC-II fibers was observed in the PARP-I group compared to the control group (PARP-I: 4774.8 μm^2^ ± 902.1, control: 3372 μm^2^ ± 887.5) (Figure 4B).

## 4. Discussion

This study demonstrates, supported by both MRI and histological data, that the PARP inhibitor Talazoparib effectively reduces muscle atrophy and fatty infiltration in retracted rotator cuff muscles in sheep. Notably, these inhibitory effects were achieved without any alterations to the muscular architecture. On the contrary, PARP inhibition resulted in a significant impact on the muscle fiber type content, leading to larger and a higher proportion of fast MHC-II fibers.

To our knowledge, the PARP inhibitor Talazoparib represents the first pharmacological therapy capable of concurrently reducing both muscle atrophy and fatty infiltration of the rotator cuff in a long-term large animal model. In contrast, the PDGFR inhibitor Imatinib only exhibits the ability to inhibit fatty infiltration [6], while the effects of tamoxifen are limited to muscle atrophy in mice [5]. Similarly, the Transforming growth factor-beta (TGF-β), small-molecule inhibitor SB431542 was able to inhibit fatty infiltration and reduce muscle wet weight loss in a mouse model [26]. However, it is important to note that investigations of muscular degeneration in mouse models are associated with methodological limitations, as both tenotomy and neurectomy are required to induce muscular degeneration of the retracted rotator cuff [5]. These procedures can elicit different physiological responses of the muscle due to the stimulus of mechanical unloading by tenotomy and denervation by neurotomy, leading to potentially dissimilar outcomes [27,28]. In contrast, the sheep tenotomy model currently used is considered more closely related to the muscular degradation observed after rotator cuff tears in humans [7]. Previous long-term large animal studies in a 16-week musculotendinous retraction model in sheep identified nandrolone as a potent inhibitor of fatty infiltration; however, it was unable to reduce muscle atrophy [7,29]. In summary, the findings from our study highlight Talazoparib as a promising therapeutic intervention capable of addressing both muscle atrophy and fatty infiltration in a clinically relevant long-term large animal model.

Muscle atrophy represents a multifaceted process characterized by changes in various signaling pathways and cellular activities, which collectively influence muscle protein dynamics. The involvement of PARP within these pathways might elucidate the observed reduction in muscle atrophy following PARP-inhibition therapies. Crucially, pathways that govern the primary cellular degradation mechanisms, such as the Ubiquitin-proteasome system and the Autophagy-lysosome system, are pivotal in the progression of muscle atrophy. Key regulatory pathways include the Forkhead box O (FoxOs)-atrogenes and the Tumor Necrosis Factor alpha (TNFa)-IκB kinase complex (IKK)–inhibitory proteins (IκB)–Nuclear Factor kappa B (NF-κB) signaling [30]. The latter has been implicated in the muscle atrophy associated with rotator cuff tears, characterized by the release of proinflammatory cytokines and the subsequent activation of NF-κB [13]. NF-κB, in turn, promotes muscle degradation through the activation of the Ubiquitin-proteasome system [31]. PARP-1, identified as a crucial co-factor for NF-κB’s transcriptional activation in vivo, plays a significant role in the inflammatory response to injury [32]. Inhibition of PARP activity is associated with a diminished inflammatory response [32].

Furthermore, the role of FoxO transcription factors in muscle wasting is well documented. Inhibition of FoxO1, 3 and 4 has been shown to completely arrest muscle loss in conditions such as fasting, hind limb suspension, immobilization, diabetes and glucocorticoid treatment [33,34,35,36]. Intriguingly, PARP-1 acts as a corepressor of FoxO1, inhibiting the expression of the cell cycle inhibitor gene p27^Kip1^ independently of its enzymatic activity [37]. Additionally, the knockdown of PARP-1 results in reduced cell proliferation, an effect that is dependent on FoxO1 function [37]. This intricate interplay between PARP-1 and FoxO1 and potential negative effect on cell proliferation underscores the complexity of effectively targeting muscle atrophy and suggests that a multifaceted approach, aimed at inhibiting several pathways, may be necessary for optimal intervention.

Moreover, additional pivotal pathways play a crucial role in the regulation of muscle growth, notably the Insulin/Insulin-like growth factor (IGF1)-Akt-Mammalian target of rapamycin (mTOR) pathway and the TGF-β/myostatin/activin/Bone Morphogenetic Protein (BMP) pathway [30]. The binding of Insulin and IGF1 to their respective receptors (IR, IGF1R) initiates a series of phosphorylation events that lead to the stimulation of protein synthesis and the inhibition of protein degradation [30]. Within this cascade, Akt and mTOR emerge as key signaling molecules, driving muscle hypertrophy [38,39,40]. Previous studies have underscored the beneficial impact of PARP inhibition on this pathway, demonstrating that PARP inhibition facilitates the activation of the Akt pathway, thereby enhancing cell survival [41]. In parallel, the regulation of skeletal muscle growth is significantly influenced by the myostatin pathway, a component of the TGFβ superfamily [42]. Myostatin, along with members of the Activin/Myostatin/TGFβ group, interacts with activin type IIB and IIA receptors located on the plasma membrane, triggering the downstream activation of Smad 2/3. This activation leads to the inhibition of both mTOR and Akt, as well as the assembly of a heterotrimeric complex with Smad4, which promotes protein degradation [43,44]. Research has revealed that PARPs intricately modulate the TGFβ pathway, fine-tuning Smad-mediated transcription through both inhibitory and stimulatory feedback mechanisms [45].

In a PARP-1 knockout mice rotator cuff tear model a significant reduction of muscle atrophy and fatty infiltration was observed in the PARP-1 deficient mice [17]. Furthermore, a significant upregulation of TGFβ, Myostatin, NF-κB and Ubiquitin-proteasome system activating factors MuRF1, as well as ligase Atrogin-1 and Ube3a, was observed in the wild-type group after 1 week, indicating an interaction of PARP-1 with the aforementioned muscle atrophy regulation pathways in rotator cuff tear-associated muscle atrophy [17]. Altogether, the interaction of PARP with several muscle atrophy associated pathways highlights the therapeutic potential of its inhibition.

Similar to muscle atrophy, fatty infiltration involves a complex array of cellular and molecular mechanisms. The precise origins of adipocytes in fatty infiltration remain elusive, but several proposed mechanisms include the proliferation of pre-existing adipocytes, differentiation from myogenic and non-myogenic mesenchymal progenitors, and the infiltration or invasion of adipocytes from surrounding tissues [6,46]. In addition to the key adipogenic transcription factors, CCAAT/enhancer-binding protein alpha (C/EBPα) and peroxisome proliferator-activated receptor gamma (PPARγ), the Wnt signaling pathway and fatty acid-binding protein 4 (FABP4) have been suggested to play roles in regulating fatty infiltration following a rotator cuff tear [47,48]. Previous research indicates that poly PARP inhibition reduces PPARγ-mediated adipogenesis [49] and serves as a crucial regulator of adipogenic differentiation [50].

PARP-I treatment resulted in a higher proportion and increased size of fast-type MHC-II muscle fibers compared to controls. Given that muscle fiber subtypes, including slow-type MHC-I and various fast-type (MHC-IIa, MHC-IIx/d, MHC-IIb) fibers, exhibit varying sensitivities to atrophy signals based on specific physiological and metabolic stimuli, muscle atrophy often involves a shift in muscle fiber type [51]. PARPs are critical cofactors in NF-κB-dependent gene transcription [52]. A previous study in a NF-κB knockout mice model demonstrated that fast muscle fiber atrophy was suppressed, and the shift from slow to fast MHC isoforms was prevented under unloading conditions in these mice [53]. The inhibition of fast muscle fiber atrophy aligns with our observations of increased size in fast MHC-II fibers following PARP-I treatment. The effect of NF-κB knockout in preventing the unloading-induced shift from MHC-I to MHC-II fibers, however, cannot be directly compared to the effects of tenotomy on muscle fibers. Previous studies indicate that tenotomy in sheep leads to a transition from MHC-II to MHC-I fibers [23]. Incorporating a neurotomy along with tenotomy appears to counteract the reduction in the area percentage of fast muscle fibers by activating a fast-contractile gene program [25]. The underlying biological mechanism by which PARP-I inhibits the tenotomy-induced shift from MHC-II to MHC-I remains to be elucidated. Moreover, previous research has indicated that atrophy of fast MHC-II fibers is a predictor of muscle weakness in elderly men [54], and a study on a clinical cohort undergoing rotator cuff reconstruction revealed that higher preoperative fast MHC-II fiber content correlated with a better clinical outcome, as measured by the Constant score, 12 months after surgery [55].

Despite its experimental design, this study has limitations. It marks the initial exploration of PARP inhibition for rotator cuff degeneration using a model without reconstruction and offers a preliminary look at the associated muscle biology. Considering animal ethics and the experimental nature of PARP-1 inhibition when this study was conducted, a control group from a previously conducted infraspinatus study was utilized. To address potential batch variability, three distinct control groups were implemented, following the identical protocol in the same facility but without the treatment. This work suggests PARP inhibition holds promise for treating musculotendinous retraction-related muscle degeneration, but further research is needed to evaluate its efficacy and biological mechanism in a reconstruction model. Additionally, the study employed a pragmatic dose of Talazoparib (0.5 mg), half the human dose, based on sheep weight. Future studies should refine the optimal dosage, timing and method of administration, as well as assess side effects.

## 5. Conclusions

In conclusion, this investigation highlights the promising efficacy of pharmacological PARP inhibition in reducing musculotendinous retraction-related muscle degeneration in the rotator cuff. MRI and histological analyses demonstrated a significant decrease in muscle atrophy and fatty infiltration within the chronically retracted infraspinatus muscles of sheep. Moreover, PARP inhibition showed potential in specifically protecting against atrophy of fast MHC-II muscle fibers. Future research to determine the optimal dosage, assess safety and elucidate the biological mechanisms is essential to fully understand the therapeutic potential of PARP inhibition in treating rotator cuff tear-induced muscle degeneration. These investigations pave the way for future clinical applications and the development of therapeutic strategies aimed at managing rotator cuff injuries in humans, thereby enhancing the outcomes of rotator cuff repair.

## Data Availability

The data presented in this study are available on request from the corresponding author. The data are not publicly available due to privacy restrictions.
